# Supplementary material for: Seroepidemiological and Molecular Survey for the Detection of SARS-CoV-2 Infection among Children in Iran, September 2020 to June 2021: 1-Year Cross-Sectional Study
Source: Microorganisms. 2023 Jun 27;11(7):1672. doi: 10.3390/microorganisms11071672 (PMC10386463; doi:10.3390/microorganisms11071672)
Supplement: Supplementary file 1 [file microorganisms-11-01672-s001.zip › microorganisms-2400061-supplementary.pdf]

Supplementary materials

Table S1. Prevalence of COVID-19 positive cases in children during autumn 2020 to spring 2021 in Teheran.[1]

	Combined results				Descriptive results for children based on ELISA and q-RT-PCR tests for each participant					
**Season**	**Total RT-qPCR Positive[2]**	**% (95% confidence intervals)**	**Total ELISA Positive[3]**	**% (95% CI)[1]**	**ELISA (+)/ qRT PCR (+)**	**% (95% CI)**	**ELISA (-)/ RT-qPCR (+)**	**% (95% CI)**	**ELISA (+)/qRT PCR (-)**	**% (95% CI)**
**Autumn 2020**	57/356	15.82 (12.24-20.22)	107/356	30.41% (25.70-35.57)	15/356	4.71% (2.78-7.87)	42/356	11.11% (8.19-14.91)	92/356	25.70% (21.23-30.74)
**Winter 2020-2021**	11/551	2.40 (1.03-5.53)	184/551	35.94% (30.58-41.67)	2/551	0.39% (0.07-2.03)	9/551	2.01% (0.77-5.16))	182/551	35.54% (30.20-41.27)
**Spring 2021**	67/610	13.93% (10.46-18.31)	207/610	33.33% (28.69-38.31)	17/610	3.71% (1.95-6.95)	50/610	10.22% (7.35-14.04)	190/610	29.62% (25.34-34.29)

1. The percentages and 95% confidence intervals are presented with population weighting considering age, gender and season.
2. Total RT-qPCR Positive represents sum of participants with PCR (+)/ELISA (-) and ELISA (+)/PCR (+) tests results.
3. Total ELISA Positive represents sum of participants with PCR (-)/ELISA (+) and ELISA (+)/PCR (+) tests results.

Table S2. Participants' characteristics by the site of sampling in Tehran, from autumn 2020 to spring 2021.

		SNAPP (%)	Tajrish Lab (%)	Vahidiye Lab (%)	Medical Lab (%)	Total (%)
Season	Autumn 2020	186 (100.00)	72 (15.06)	34 (20.99)	63 (9.12)	355 (23.40)
	Winter 2020	0 (0.00)	284 (59.41)	90 (55.56)	176 (25.47)	550 (36.26)
	Spring 2021	0 (0.00)	122 (25.52)	38 (23.46)	451 (65.27)	611 (40.28)
	summer 2021	0 (0.00)	0 (0.00)	0 (0.00)	0 (0.00)	0 (0.00)
	Missing	0 (0.00)	0 (0.00)	0 (0.00)	1 (0.14)	1 (0.07)
Geographic location	Northern	39 (24.52)	37 (16.66)	1 (1.07)	9 (3.54)	86 (11.81)
	Southern	26 (16.35)	94 (42.34)	30 (32.25)	92 (36.22)	242 (33.24)
	Western	9 (5.66)	23 (10.36)	6 (6.45)	14 (5.51)	52 (7.14)
	Eastern	53 (33.33)	49 (22.07)	49 (52.68)	101 (39.76)	252 (34.61)
	Central	32 (20.12)	19 (8.55)	7 (7.52)	38 (14.96)	96 (13.18)
	Missing	27 (14.51)	0 (0.00)	0 (0.00)	762 (75.00)	789 (52.01)
Gender	female	74 (39.78)	133 (27.82)	46 (28.40)	230 (33.29)	483 (31.84)
	male	109 (58.60)	337 (70.50)	115 (70.99)	458 (66.28)	1019 (67.17)
	Missing	3 (1.61)	8 (1.67)	1 (0.62)	3 (0.43)	15 (0.99)
Age	<=4y	60 (32.26)	259 (54.18)	87 (53.70)	183 (26.48)	589 (38.83)
	5-9y	72 (38.71)	105 (21.97)	30 (18.52)	84 (12.16)	291 (19.18)
	10-14y	54 (29.03)	29 (6.07)	21 (12.96)	34 (4.92)	138 (9.10)
	Missing	0 (0.00)	85 (17.78)	24 (14.81)	390 (56.44)	499 (32.89)
Smoker in house	yes	44 (23.66)	69 (14.44)	35 (21.60)	121 (17.51)	269 (17.73)
	No	106 (56.99)	390 (81.59)	123 (75.93)	482 (69.75)	1101 (72.58)
	Missing	36 (19.35)	19 (3.97)	4 (2.47)	88 (12.74)	147 (9.69)
Socioeconom ic status	high	0 (0.00)	3 (0.63)	3 (1.85)	5 (0.72)	11 (0.73)
	mid	14 (7.53)	210 (43.93)	94 (58.02)	300 (43.42)	618 (40.74)
	low	0 (0.00)	226 (47.28)	53 (32.72)	238 (34.44)	517 (34.08)
	Missing	172 (92.47)	39 (8.16)	12 (7.41)	148 (21.42)	371 (24.46)
Family Members	2-3	74 (39.78)	179 (37.45)	70 (43.21)	277 (40.09)	600 (39.55)
	4	85 (45.70)	194 (40.59)	65 (40.12)	264 (38.21)	608 (40.08)
	>=5	25 (13.44)	86 (17.99)	24 (14.81)	122 (17.66)	257 (16.94)
	Missing	2 (1.08)	19 (3.97)	3 (1.85)	28 (4.05)	52 (3.43)
Contact with a COVID patient during last 4 weeks	yes	101 (54.30)	10 (2.09)	18 (11. 11)	62 (8.97)	191 (12.59)
	no	83 (44.62)	414 (86.61)	127 (78.40)	493 (71.35)	1117 (73.63)

"Continued"

		SNAPP (%)	Tajrish Lab (%)	Vahidiye Lab (%)	Medical Lab (%)	Total (%)
	unknown	1 (0.54)	40 (8.37)	14 (8.64)	54 (7.81)	109 (7.19)
	Missing	1 (0.54)	14 (2.93)	3 (1.85)	82 (11.87)	100 (6.59)
Symptomatic (fever or cough or diarrhea)/ Asymptomatic	Asymptomatic	115 (61.83)	419 (87.66)	145 (89.51)	468 (67.73)	1147 (75.61)
	Symptomatic	70 (37.63)	44 (9.21)	12 (7.41)	137 (19.83)	263 (17.34)
	Missing	1 (0.54)	15 (3.14)	5 (3.09)	86 (12.45)	107 (7.05)
Sore throat	yes	45 (24.19)	16 (3.35)	5 (3.09)	59 (8.54)	125 (8.24)
	no	138 (74.19)	431 (90.17)	145 (89.51)	496 (71.78)	1210 (79.76)
	Missing	3 (1.61)	31 (6.49)	12 (7.41)	136 (19.68)	182 (12.00)
Fatigue	yes	36 (19.35)	11 (2.30)	8 (4.94)	62 (8.97)	117 (7.71)
	no	148 (79.57)	436 (91.21)	143 (88.27)	498 (72.07)	1225 (80.75)
	Missing	2 (1.08)	31 (6.49)	11 (6.79)	131 (18.96)	175 (11.54)
Fever >38 ^{o}C	yes	36 (19.35)	18 (3.77)	9 (5.56)	85 (12.30)	148 (9.76)
	no	147 (79.03)	439 (91.84)	141 (87.04)	483 (69.90)	1210 (79.76)
	Missing	3 (1.61)	21 (4.39)	12 (7.41)	123 (17.80)	159 (10.48)
Cough	yes	41 (22.04)	20 (4.18)	6 (3.70)	63 (9.12)	130 (8.57)
	no	144 (77.42)	431 (90.17)	146 (90.12)	498 (72.07)	1219 (80.36)
	Missing	1 (0.54)	27 (5.65)	10 (6.17)	130 (18.81)	168 (11.07)
Rhinorrhea	yes	42 (22.58)	17 (3.56)	9 (5.56)	55 (7.96)	123 (8.11)
	no	143 (76.88)	435 (91.00)	143 (88.27)	504 (72.94)	1225 (80.75)
	Missing	1 (0.54)	26 (5.44)	10 (6.17)	132 (19.10)	169 (11.14)
Stomachache	yes	26 (13.98)	26 (5.44)	17 (10.49)	69 (9.99)	138 (9.10)
	no	155 (83.33)	427 (89.33)	136 (83.95)	490 (70.91)	1208 (79.63)
	Missing	5 (2.69)	25 (5.23)	9 (5.56)	132 (19.10)	171 (11.27)
Headache	yes	33 (17.74)	13 (2.72)	6 (3.70)	31 (4.49)	83 (5.47)
	no	144 (77.42)	440 (92.05)	146 (90.12)	518 (74.96)	1248 (82.27)
	Missing	9 (4.84)	25 (5.23)	10 (6.17)	142 (20.55)	186 (12.26)
Nausea/vomiting	Yes	19 (10.22)	10 (2.09)	5 (3.09)	47 (6.80)	81 (5.34)
	No	166 (89.25)	441 (92.26)	147 (90.74)	513 (74.24)	1267 (83.52)
	Missing	1 (0.54)	27 (5.65)	10 (6.17)	131 (18.96)	169 (11.14)
Diarrhea	Yes	15 (8.06)	11 (2.30)	5 (3.09)	46 (6.66)	77 (5.08)
	No	169 (90.86)	436 (91.21)	141 (87.04)	511 (73.95)	1257 (82.86)
	Missing	2 (1.08)	31 (6.49)	16 (9.88)	134 (19.39)	183 (12.06)

“Continued”

		SNAPP (%)	Tajrish Lab (%)	Vahidiye Lab (%)	Medical Lab (%)	Total (%)
Wheeze	Yes	9 (4.84)	6 (1.26)	1 (0.62)	25 (3.62)	41 (2.70)
	no	176 (94.62)	444 (92.89)	151 (93.21)	524 (75.83)	1295 (85.37)
	Missing	1 (0.54)	28 (5.86)	10 (6.17)	142 (20.55)	181 (11.93)
Dyspnea	yes	4 (2.15)	5 (1.05)	1 (0.62)	12 (1.74)	22 (1.45)
	no	181 (97.31)	447 (93.51)	151 (93.21)	535 (77.42)	1314 (86.62)
	Missing	1 (0.54)	26 (5.44)	10 (6.17)	144 (20.84)	181 (11.93)
Myalgia	yes	24 (12.90)	0 (0.00)	0 (0.00)	0 (0.00)	24 (1.58)
	no	154 (82.80)	439 (91.84)	144 (88.89)	520 (75.25)	1257 (82.86)
	Missing	8 (4.30)	39 (8.16)	18 (11.11)	171 (24.75)	236 (15.56)
Chest pain	yes	4 (2.15)	3 (0.63)	2 (1.23)	10 (1.45)	19 (1.25)
	no	171 (91.94)	448 (93.72)	148 (91.36)	537 (77.71)	1304 (85.96)
	Missing	11 (5.91)	27 (5.65)	12 (7.41)	144 (20.84)	194 (12.79)
Other respiratory symptoms	yes	1 (0.54)	3 (0.63)	0 (0.00)	6 (0.87)	10 (0.66)
	no	176 (94.62)	447 (93.51)	152 (93.83)	537 (77.71)	1312 (86.49)
	Missing	9 (4.84)	28 (5.86)	10 (6.17)	148 (21.42)	195 (12.85)
Change in smell or taste	yes	0 (0.00)	3 (0.63)	3 (1.85)	3 (0.43)	9 (0.59)
	no	11 (5.91)	343 (71.76)	78 (48.15)	445 (64.40)	877 (57.81)
	unknown	2 (1.08)	67 (14.02)	26 (16.05)	66 (9.55)	161 (10.61)
	Missing	173 (93.01)	65 (13.60)	55 (33.95)	177 (25.62)	470 (30.98)

Figure S1. Reported COVID-19 cases from February 2020 to October 2022 in Iran.

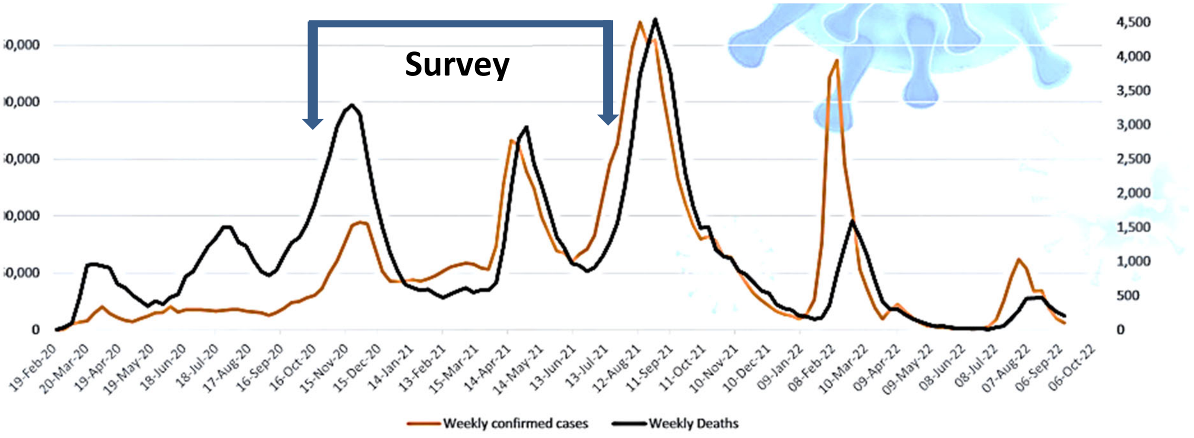

Source: http://www.emro.who.int/iran/information-resources/COVID-19-situation-reports.html; Number 846, 10 September 2022. The survey was conducted in Tehran between 19 September 2020 and 21 June 2021.
